# Novel Chikungunya Vaccine Candidate with an IRES-Based Attenuation and Host Range Alteration Mechanism

**DOI:** 10.1371/journal.ppat.1002142

**Published:** 2011-07-28

**Authors:** Kenneth Plante, Eryu Wang, Charalambos D. Partidos, James Weger, Rodion Gorchakov, Konstantin Tsetsarkin, Erin M. Borland, Ann M. Powers, Robert Seymour, Dan T. Stinchcomb, Jorge E. Osorio, Ilya Frolov, Scott C. Weaver

**Affiliations:** 1 Institute for Human Infections and Immunity, Sealy Center for Vaccine Development, and Department of Pathology, University of Texas Medical Branch, Galveston, Texas, United States of America; 2 Inviragen Inc, Madison, Wisconsin and Fort Collins, Colorado, United States of America; 3 Department of Pathobiological Sciences, School of Veterinary Medicine, University of Wisconsin, Madison, Wisconsin, United States of America; 4 Division of Vector Borne Infectious Diseases, Centers for Disease Control and Prevention, Fort Collins, Colorado, United States of America; 5 Department of Microbiology, University of Alabama, Birmingham, Alabama, United States of America; University of North Carolina at Chapel Hill, United States of America

## Abstract

Chikungunya virus (CHIKV) is a reemerging mosquito-borne pathogen that has recently caused devastating urban epidemics of severe and sometimes chronic arthralgia. As with most other mosquito-borne viral diseases, control relies on reducing mosquito populations and their contact with people, which has been ineffective in most locations. Therefore, vaccines remain the best strategy to prevent most vector-borne diseases. Ideally, vaccines for diseases of resource-limited countries should combine low cost and single dose efficacy, yet induce rapid and long-lived immunity with negligible risk of serious adverse reactions. To develop such a vaccine to protect against chikungunya fever, we employed a rational attenuation mechanism that also prevents the infection of mosquito vectors. The internal ribosome entry site (IRES) from encephalomyocarditis virus replaced the subgenomic promoter in a cDNA CHIKV clone, thus altering the levels and host-specific mechanism of structural protein gene expression. Testing in both normal outbred and interferon response-defective mice indicated that the new vaccine candidate is highly attenuated, immunogenic and efficacious after a single dose. Furthermore, it is incapable of replicating in mosquito cells or infecting mosquitoes in vivo. This IRES-based attenuation platform technology may be useful for the predictable attenuation of any alphavirus.

## Author Summary

Chikungunya virus (CHIKV) is a mosquito-borne alphavirus that has reemerged since 2004 to cause millions of cases of severe and often persistent arthralgia. Because no licensed vaccine exists to prevent this disease, we utilized an attenuation approach to produce a live CHIKV vaccine candidate that elicits a robust, protective immune response yet causes no detectable disease in mice. It is also incapable of infecting mosquito vectors, an important safety feature for a live virus vaccine that may be used in nonendemic locations to immunize travelers or laboratory personnel. This vaccine approach, which exploits the attenuating effect of altering the expression of the alphavirus structural proteins with a picornavirus IRES, may be broadly applicable to other alphaviruses that cause important febrile diseases as well as encephalitis.

## Introduction

Chikungunya (CHIK) virus (CHIKV) is a reemerging arboviral pathogen that has recently caused explosive urban outbreaks involving millions of persons in Africa and Asia. The virus was first isolated from a human in Tanzania in 1953 during a major epidemic [1], and derives its name from a Makonde word meaning “that which bends up,” which describes the posture observed in afflicted persons. CHIKV typically causes a febrile illness and severe joint pain, which is clinically similar to dengue fever. These 2 viruses also share similar endemic distributions in the Eastern Hemisphere, resulting in many CHIKV cases being misdiagnosed when laboratory testing is not available [2]. Large CHIK outbreaks were described during the 1950's and 60's in India and Southeast Asia [3], [4]. However, it was not until 2005 that CHIKV gained widespread public attention due to massive outbreaks on islands of the Indian Ocean [5] and later in India [6] and Southeast Asia [7]. In total, several million persons have been affected [8], [9]. On the Island of Reunion alone, ca. 300,000 persons or one-third of the population was affected [10]. Another factor driving the resurgence of interest in CHIK is the detection of occasional fatal cases, which were not documented before. Previously, individuals who became severely ill typically presented with hemorrhagic manifestations and occasionally shock [11], [12], [13]. However, the recent outbreaks have been linked to thousands of deaths in Reunion and India due to neurologic disease [14], [15], [16].

CHIKV exists in two transmission cycles: an enzootic or sylvatic cycle and an endemic/epidemic urban cycle. The African sylvatic cycle likely involves several arboreal *Aedes* mosquitoes as vectors and nonhuman primates as reservoir/amplifying hosts [17]. African outbreaks occur from direct enzootic spillover or when CHIKV is introduced into an urban areas inhabited by the anthropophilic mosquito vector, *Aedes aegypti*. [17], [18]. More permanent endemic/epidemic transmission cycles were established when the virus was introduced into Asia ca. 1950, and into the Indian Ocean region, India and then Southeast Asia since 2005 [19]. A mutation in the E1 envelope glycoprotein gene that results in an A226V amino acid substitution dramatically increased the infectivity of some epidemic strains for an alternative urban vector, *Ae. albopictus* [8], [20]. The nearly ubiquitous distribution of *Ae. aegypti*, and the expanding distribution of *Ae. albopictus* into tropical and temperate regions of both hemispheres has raised concern that CHIKV may spread outside of its previous endemic region into the Western Hemisphere and Europe. The latter scenario was realized in 2007 during a small epidemic in Italy [21] and during autochthonous transmission in southern France during 2010 (ProMED archive 20100926.3495).

**Figure 1:**
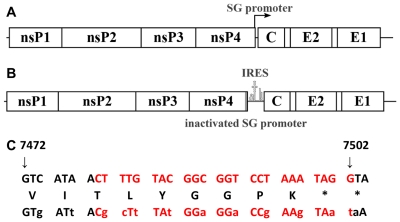
Genetic organization of wild-type CHIKV and the sequence of the subgenomic promoter. A. Cartoon showing the locations of the 2 open reading frames encoding the nonstructural proteins and the subgenomic message encoding the capsid (C) as well as the envelope glycoproteins E2 and E1. B. Inactivation of the subgenomic promoter and insertion of the encephalomyocarditis internal ribosome entry site (IRES) to drive expression of the structural proteins from the genomic message. C. Wild-type subgenomic promoter (above) and inactivated promoter sequence (below) with synonymous mutations in lower-case. Deduced amino acid sequence is between nucleotide sequences. Red letters represent the subgenomic promoter region.

CHIKV belongs to the family *Togaviridae*, genus *Alphavirus*, whose members are enveloped virions that contain a positive sense, single stranded, RNA genome of ∼12 kb. The genome encodes 4 non-structural proteins (nsP1-4) and 3 major structural proteins (Capsid, E1, and E2 envelope glycoproteins)(Fig. 1A). During replication, two distinct RNA's are produced: the genomic and subgenomic RNAs. A negative sense template RNA is also produced. The nonstructural polyprotein open reading frame (ORF) is translated via a cap-dependant mechanism from the genomic RNA, whereas the structural protein gene ORF is translated from the subgenomic RNA, also in a cap-dependent manner. The subgenomic RNA is transcribed late during infection from its promoter, which is found in the 3′ end of the nsP4 gene [22].

There is no licensed vaccine or therapeutic CHIK, so outbreaks can only be controlled by preventing the exposure of people to infected mosquito vectors. Scientists at the Walter Reed Army Institute of Research produced an investigational vaccine called 181/clone 25 (hereafter called 181/25) during the 1980s. This live-attenuated strain was generated via serial plaque-to-plaque passages of a wild-type Thai CHIKV strain using MRC-5 cells [23]. The virus is attenuated in both rodents and non-human primates and is highly immunogenic in humans. However, during phase II trials, strain 181/25 caused mild, transient arthralgia in 5 of 59 vaccinees [24]. Also, strain 181/25 can be transmitted experimentally by the natural mosquito vector, *Ae. aegypti* [25].

To be effective in resource-limited nations that are endemic for CHIK as well as to combat an epidemic, an ideal CHIK vaccine would induce rapid and long-lived immunity after a single dose, have a low risk of reactogenicity and reversion to virulence, and be inexpensive. Vaccines against arboviral diseases should also have a low risk of transmission from vaccinated persons via mosquitoes in the event that viremia occurs, especially those used in non-endemic regions. Although replication-defective vaccine candidates have been described that emphasize safety [26], [27], [28], none has been shown to induce rapid or long-lived immunity after a single dose, and some may be expensive to produce. In contrast, live-attenuated vaccines like the yellow fever 17D vaccine [29] have been spectacularly successful in preventing disease in developing tropical regions.

To generate a safer and more effective live-attenuated CHIK vaccine that meets the criteria outlined above, we previously produced and tested a series of chimeric alphaviruses containing either Venezuelan equine encephalitis virus (VEEV), eastern equine encephalitis or Sindbis virus non-structural protein genes along with the CHIKV structural protein genes [30]. These vaccines produce robust neutralizing antibody (Ab) responses and provide complete protection against disease after CHIKV challenge. However, some residual ability to infect potential mosquito vectors remains, and attenuation is dependent on an intact murine interferon response (SCW, unpublished). To overcome these limitations, we developed a new attenuation strategy and conducted proof-of-principle studies with another alphavirus, VEEV vaccine strain TC-83. Both attenuation and elimination of mosquito infectability relied on the inactivation of the subgenomic promoter, and addition of a encephalomyocarditis virus (EMCV) internal ribosome entry sequence (IRES) to drive translation of the structural protein genes [31]. Chimeric alphaviruses incorporating the IRES element have also been generated as vaccine candidates [32]. The EMCV IRES also mediates inefficient translation in arthropod cells [33], rendering these mutants unable to infect mosquitoes. However, starting with the attenuated TC-83 strain, the IRES-based attenuation resulted in inadequate immunogenicity and the lack of a neutralizing Ab response.

Here, we implemented this IRES-based vaccine design for CHIKV using a cDNA clone generated from the wild-type La Reunion strain [34]. Testing of this novel vaccine candidate in several murine models indicated that it is highly attenuated, even in the absence of an intact murine IFN response, is immunogenic and efficacious in preventing CHIK disease, and is unable to infect mosquitoes.

## Results

### Production of recombinant CHIKV/IRES vaccine candidate

**Figure 2:**
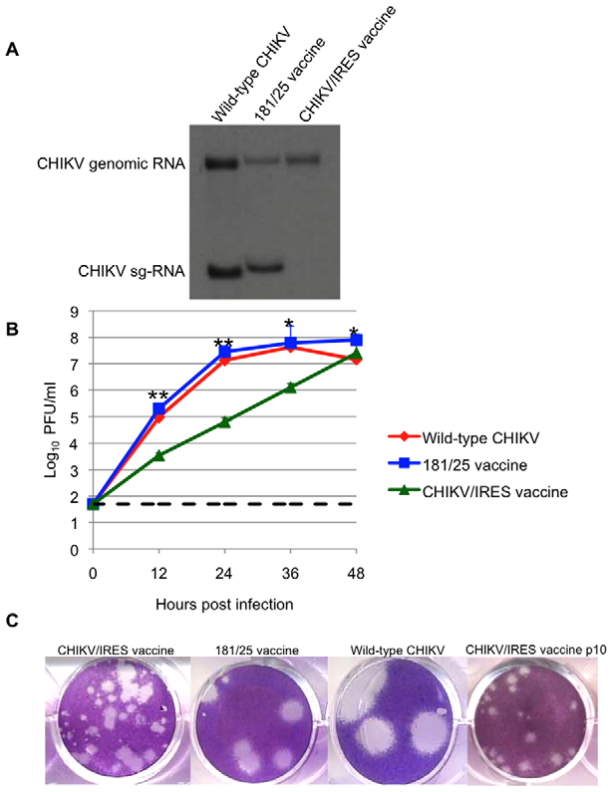
Replication of CHIKV/IRES in vitro. A. Viral RNA present in Vero cells 22 hours after infection with CHIKV/IRES and CHIKV strains LR and 181/25. Slight differences in genomic and subgenomic RNA sizes of wt-LR strain and 181/25 reflect differences in untranslated sequence lengths. B. Replication kinetics of vaccine strains CHIKV/IRES and 181/25, as well as wt CHIKV in Vero cells after infection at a multiplicity of 0.1 PFU/cell. C. Plaque morphology of vaccine strains CHIKV/IRES, CHIKV/IRES Vero p10, and 181/25, as well as wt-CHIKV 3 days after infection of Vero cells. *  = p<0.05; **  = p<0.001.

The CHIKV/IRES vaccine candidate was generated in cDNA form using standard recombinant DNA techniques using the IRES-based attenuation strategy tested previously in TC-83 [31]. The IRES element was amplified from the original TC-83/IRES construct including the first 4 codons from the EMCV sequence that were previously shown to have no effect on viral replication [31]. The IRES sequence was placed directly downstream from the subgenomic promoter of the La Reunion (LR) CHIKV infectious cDNA clone (Fig. 1B) [34]. The subgenomic promoter was inactivated using 13 synonymous mutations to preserve the wild-type amino acid sequence of nsP4 (Fig. 1C). The resultant virus, rescued by electroporation of in vitro-transcribed RNA into Vero cells, contained a non-functional subgenomic promoter as indicated by the absence of subgenomic RNA within infected cells (Fig. 2A).

Titers of CHIKV/IRES collected 30 h after electroporation were 6×10^6^ plaque forming units (PFU)/ml, in comparison to titers of 1.1x10^7^ for wild-type (wt) CHIKV strain LR. To assess replication kinetics, viruses derived from the electroporation were compared after infection of Vero cells. The CHIKV/IRES replicated more slowly than 181-25 or wt-CHIKV, requiring 48 hours at 37°C to reach a peak titer of 2.5×10^7^ PFU/ml. Strain 181-25 replicated almost to peak titer within 24 hours and reached 7.9×10^7^ PFU/ml. The wt-CHIKV also replicated close to its peak titer by 24 hours and reached 4.2×10^7^ (Fig. 2B). Unlike wt-CHIKV, which produced visible plaques within 48 hours of Vero cell infection, the CHIKV/IRES plaques were not readily visible before 3 days of incubation at 37°C. CHIKV/IRES plaques were 0.5–2 mm in diameter, whereas vaccine strain 181/25 produced 2–4 mm and wt CHIKV produced ca. 6 mm plaques under 0.4% agarose at 3 days post infection (Fig. 2C).

### Stability following cell culture passages

To assess phenotypic and genetic stability, CHIKV/IRES was passaged 10 times in Vero cells at 37°C using a multiplicity of infection of 0.1 PFU/cell. The plaque morphology remained heterogeneous but consistent after the 10 passages (Fig. 2C). Sequencing of reverse transcription-polymerase chain reaction (RT-PCR) amplicons covering the entire genome revealed no consensus mutations aside from the presence of adenine insertions within a poly-A track of the IRES element itself. Plaque purified clones were sequenced through the IRES to determine the frequency of these mutations; 8 of 10 plaque clones examined had 7 As like the original cDNA clone and the 10^th^ passage consensus sequence. However, 3 biological clones had up to 17 As in this region. These differences in sequence showed no obvious correlation with plaque size (data not shown).

CHIKV/IRES was also blind-passaged 5 times in C6/36 *Ae. albopictus* cells and the presence of virus was detected by the ability to produce cytopathic effects (CPE) on Vero cells and by RT-PCR amplification. Virus was detected only after the first passage, which presumably reflected residual virus that could not be washed from the cells after inoculation, and was not detected thereafter (data not shown). In contrast, the wt-CHIKV strain replicated in the mosquito cells throughout the passages, with titers ranging from 3–5×10^7^ PFU/ml.

### Attenuation in infant CD-1 mice

**Figure 3:**
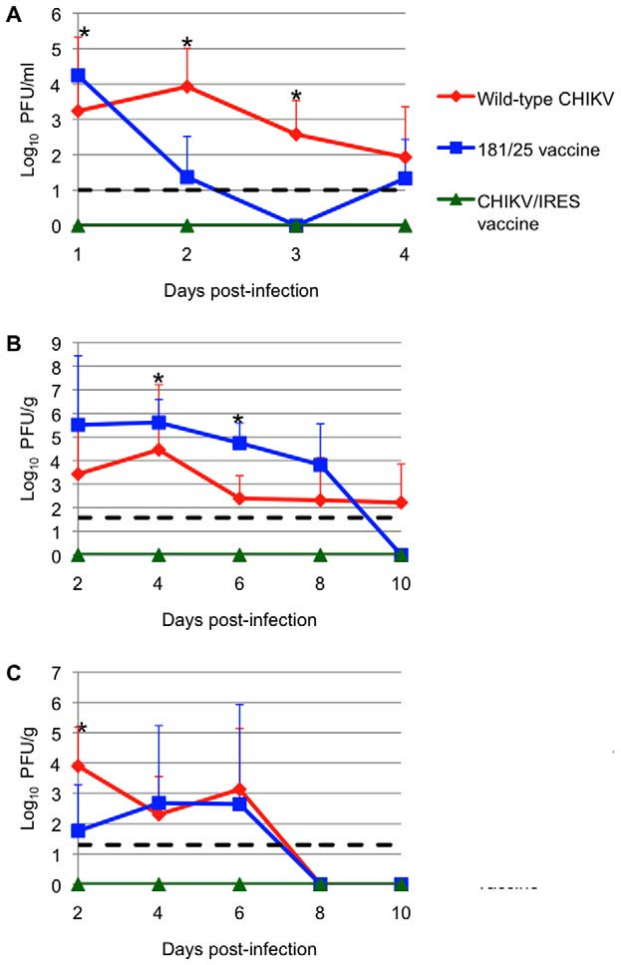
Vaccination of 6-day-old CD-1 mice after with 10^6^ PFU of wt-CHIKV or vaccine candidates 181/25 or CHIKV/IRES. A: viremia; B: knee tissue; C: brain. Dashed line shows limit of detection for the plaque assay. Bars indicate standard deviations. *  = p<0.05.

Infant outbred CD1 mice develop CHIK disease similar in many ways to that seen in humans [35]. We therefore used this model to evaluate the attenuation of our CHIKV/IRES vaccine candidate. Cohorts (N = 3) of 6-day-old CD1 mice were injected subcutaneously (SC) with 10^5 ^PFU (A high dose to increase sensitivity to detect virulence) of strains 181/25, wt LR, or CHIKV/IRES, and were sacrificed on days 2, 4, 6, and 8 to compare viral loads. Blood, brain, and leg tissue (including the knee) were collected and titrated for infectious virus. The CHIKV/IRES strain produced no detectable virus in any tissue measured throughout the sampling period. In contrast, both vaccine strain 181/25 and wt CHIKV produced measurable and significantly higher viremia through day 4 (p<0.05)(Fig. 3A). Surprisingly, vaccine strain 181/25 produced higher viral titers in leg tissue than wt strain LR, and both wt-CHIKV and 181/25 leg titers were significantly higher than those of CHIKV/IRES (p<0.05)(Fig. 3B). The wt CHIKV strain produced significantly higher brain titers than either vaccine strain on day 2 (p<0.05)(Fig. 3C). These results indicated that CHIKV/IRES is strongly attenuated in the baby mouse model.

### Attenuation in A129 mice

**Figure 4:**
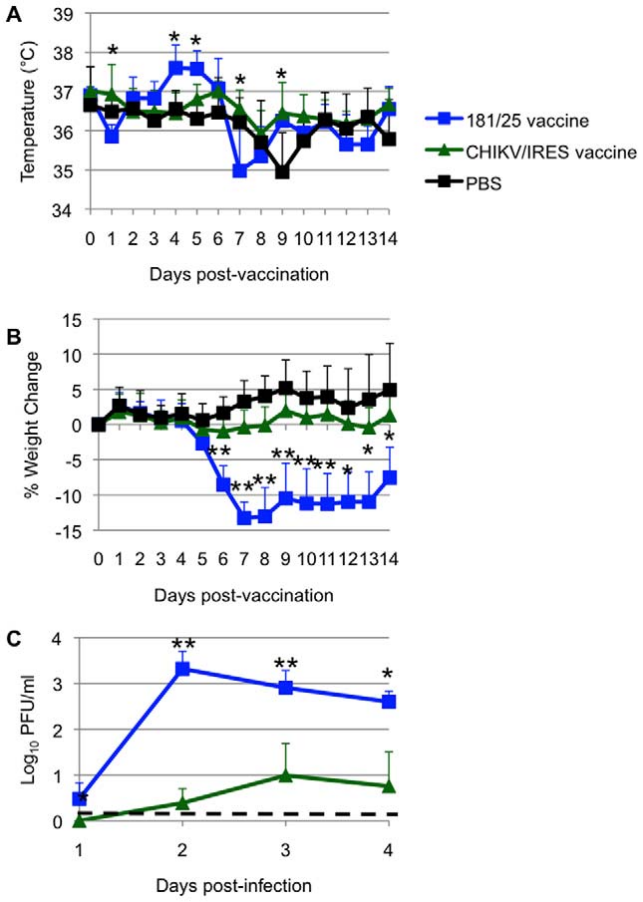
Vaccination of 10-week-old A129 mice. Mice were inoculated intradermally with 10^4^ PFU of CHIKV/IRES or 181/25, or after sham infected with PBS. A. Temperature. B. Weights. C. Viremia determined using qRT-PCR. Bars indicate standard deviations. N = 7 for CHIKV/IRES and N = 4 for strain 181/25. *  =  p<0.05. and **  =  p<0.001.

Another murine model for CHIKV pathogenesis is the A129 mouse, which lacks functional type I interferon receptors. This model has the advantage of producing disease in adult animals, thus permitting efficacy testing using wt-CHIKV challenge [36]. Cohorts of 10-week-old homozygous A129 mice were injected intradermally in the footpad with 10^4 ^PFU (more than 100 LD_50_ for wt-CHIKV) of either CHIKV/IRES (N = 7) or 181/25 (N = 4), and negative controls were sham (PBS)-infected (N = 6). Mice infected with the CHIKV/IRES vaccine showed no visible signs of illness (weight loss, temperature change, ruffling of fur or hunched posture) during 14 days of observation. Mice receiving strain 181/25 exhibited significant hyperthermia from day 4–5, and also showed significant weight loss on day 6 post vaccination (p<0.05), compared to the more constant temperatures and weight increases observed in the mice receiving CHIKV/IRES (Figs. 4A and B). Both CHIKV/IRES and 181/25 produced viremia in A129 mice, but mean titers were consistently lower for CHIKV/IRES (Fig. 4C). These data suggested greater attenuation of CHIKV/IRES compared with181/25.

**Figure 5:**
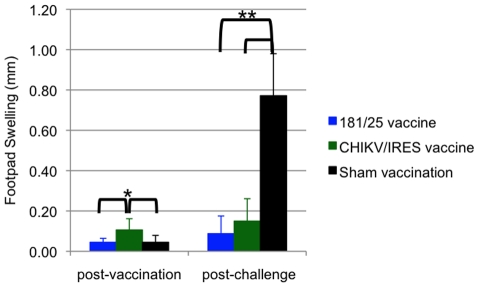
Foot swelling for 10-week-old A129 mice after intradermal vaccination with 10^4^ PFU of CHIKV/IRES or 181/25. The mice were challenged 30 days later with wt-CHIKV and a post-challenge measurement was taken 48 hours after. Foot thickness was measured with a caliper as the vertical height of the hind feet at the balls. Bars indicate standard deviations. N = 7 for CHIKV/IRES and N = 4 for strain 181/25. *  = p<0.05. and **  = p<0.001.

Another sign of disease monitored in A129 mice was swelling of the feet. For this measurement, mice were vaccinated as described above and subsequently challenged with 100 PFU of wt-CHIKV one month post-vaccination in the same foot as the vaccination site. Two days after vaccination or challenge, the vertical heights of the hind feet were measured using a caliper at the balls. PBS and 181/25 vaccination produced small and similar amounts of swelling (ca. 0.05 mm), while CHIKV/IRES vaccination produced slightly greater but still minimal swelling of 0.1 mm (Fig. 5). Sham-vaccinated mice that were challenged showed a strong inflammatory response with a mean increase of 0.8 mm in footpad thickness. In contrast, both vaccines protected significantly against swelling (p<0.001) with no significant difference between 181/25 and CHIKV/IRES.

**Figure 6:**
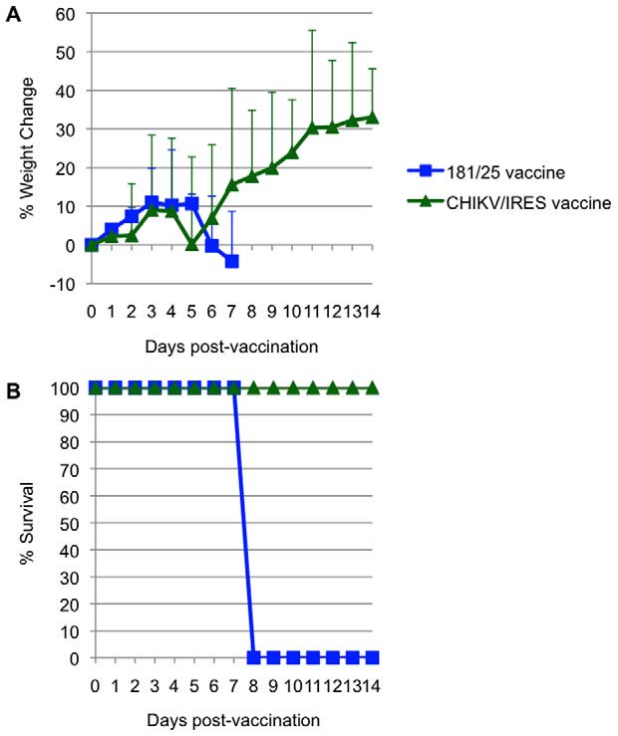
Virulence for 3-week-old A129 mice for strains 181/25 and CHIKV/IRES. A. Weight post-vaccination. B. Survival post-vaccination.

Attenuation of the 2 vaccine candidates was also compared by infection of 3-week-old A129 mice. Cohorts of 5 were injected intradermally with 10^4^ PFU (>100 LD_50_ for wt-CHIKV) of either 181/25 or CHIKV/IRES. The mice were monitored for weight and survival. There was no significant difference between the weight changes of the two cohorts (Fig. 6A). All animals that received the 181/25 vaccine died or had to be euthanized by day 8 (Fig. 6B). In contrast, none of the animals inoculated with the CHIKV/IRES vaccine showed any signs of illness and all survived to the end of the study 14 days after infection.

### Immunogenicity and efficacy in A129 mice

**Figure 7:**
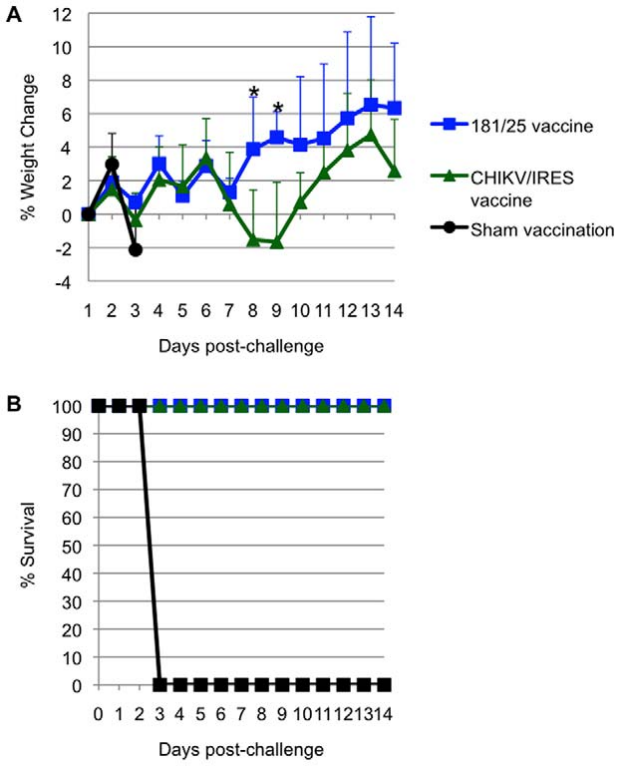
Weight and survival in 10-week-old A129 vaccinated with CHIKV/IRES, 181/25, or PBS, then challenged with wt- CHIKV (100 PFU). A. Weight post-challenge. B. Survival post-challenge. *  = p value<0.05.

All A129 mice that received vaccine candidates 181/25 (N = 4) or CHIKV/IRES (N = 7) at a dose of 10^4^ PFU seroconverted. All titers measured 35 days after vaccination exceeded 320, except for one mouse immunized with strain 181/25 that had a PRNT_80_ titer of 160. None of the animals that received CHIKV/IRES or 181/25 showed a significant temperature change (data not shown) or any other signs of illness (as described above) after challenge with 100 PFU of wt CHIKV, and all survived until day 14 after challenge, when the study was terminated. Mice vaccinated with 181/25 exhibited stable or slightly increasing weight after challenge, while the CHIKV/IRES-vaccinated mice lost some weight on days 8 and 9 post challenge, then recovered. In sharp contrast, sham-vaccinated animals rapidly lost weight before succumbing to infection (Fig. 7A and B). Both vaccines were significantly (Kaplan-Meier, p<0.05) and equally efficacious in preventing fatal CHIK in the A129 model.

**Figure 8:**
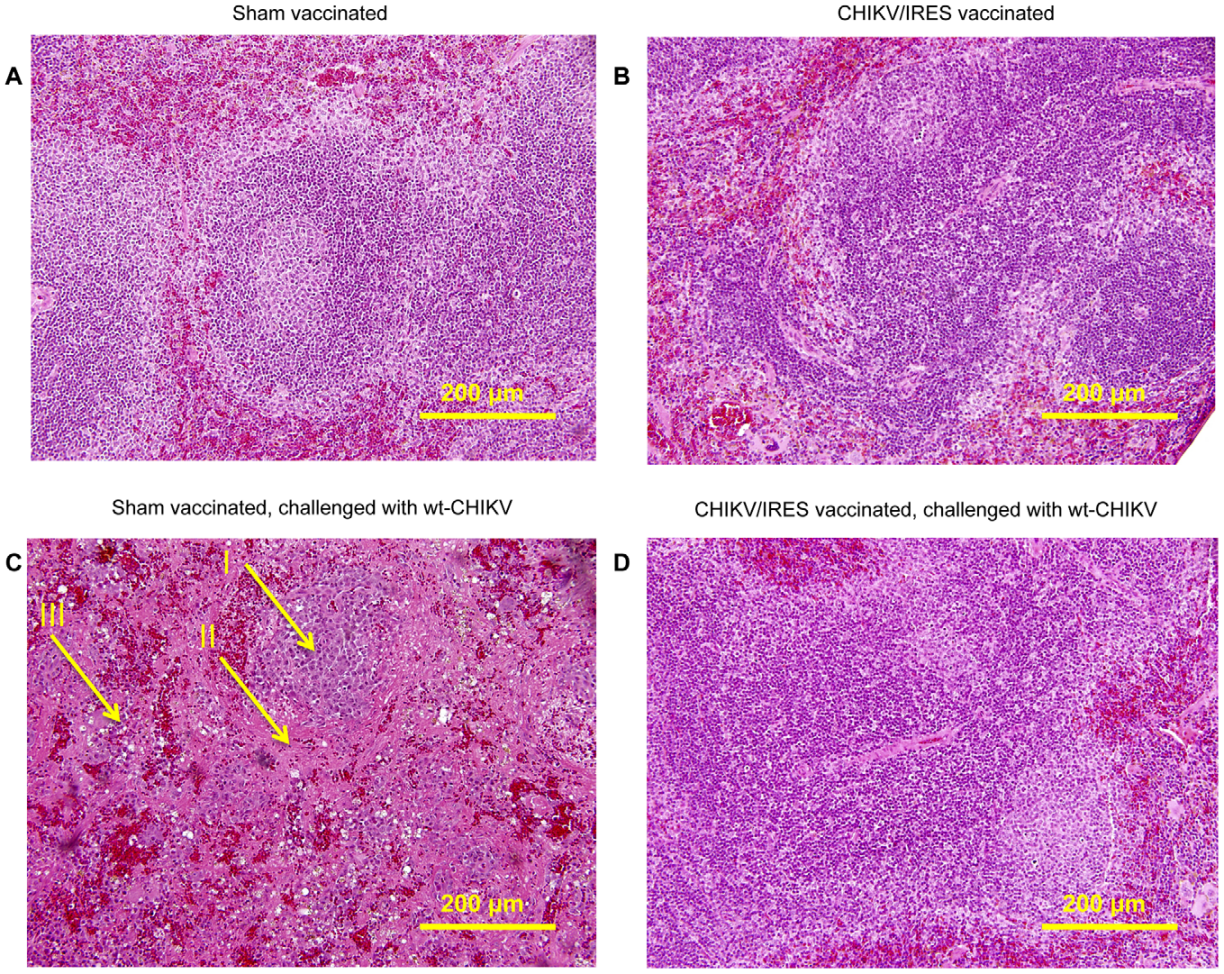
Representative splenic histopathology of A129 mice post-vaccination and –challenge, 20X magnification. A. PBS-vaccinated animal 4 days post-vaccination. B. CHIKV/IRES-vaccinated animal, 4 days post-vaccination. C. PBS-vaccinated animals, 4 days post-challenge; I: center of remnant lymphoid follicle. II: proteinacious debris. III: monocytoid cells. D. CHIKV/IRES vaccinated animals, 4 days post-challenge.

The ability of the CHIKV/IRES vaccine candidate to protect against disease was also measured histopathologically in A129 mice after wt-CHIKV challenge. Because unprotected mice die before muscle or joint lesions develop (SCW, RS, unpublished), we examined the spleen, where earlier lesions occur. Cohorts of three 8–10-week-old A129 mice were vaccinated intradermally in the footpad with either 10^4^ PFU of CHIKV/IRES or were sham-vaccinated with PBS. One mouse from each cohort was sacrificed 4 days post vaccination, and the remaining 2 mice were challenged with 100 PFU of wt-CHIKV at 26 days post-vaccination, then sacrificed 4 days post-challenge. The spleens of the sham-vaccinated mice challenged with CHIK-LR exhibited severe necrosis with markedly reduced numbers of small lymphocytes in the mantle and marginal zones. Only the central portion of the remnant lymphoid follicle remained. In addition, monocytoid cells with abundant eosinophilic cytoplasm in the interfollicular region were observed (Fig. 8C). In contrast, the spleens of animals receiving the vaccine as well as CHIKV/IRES-vaccinated mice challenged with wt-CHIKV (Fig. 8B & D) exhibited normal splenic architecture with intact lymphoid follicles and appropriate quantities of white and red pulp. The key histopathologic finding was the absence of any necrosis in the CHIK/IRES-vaccinated animals, when compared to the sham-vaccinated mice.

### Duration of immunity in A129 mice

**Figure 9:**
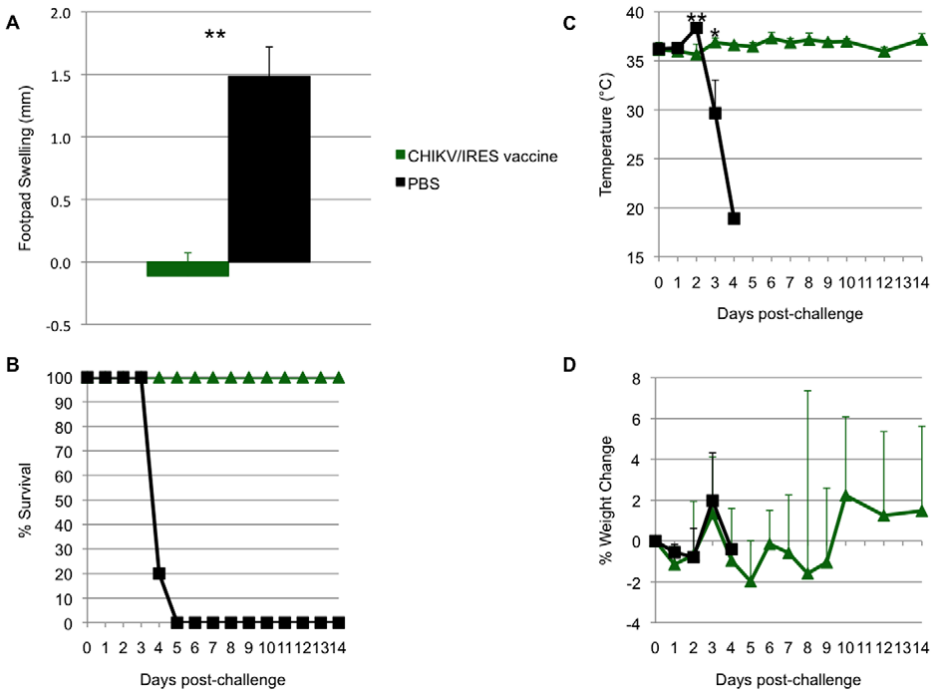
Duration of immunity in A129 mice. Mice were vaccinated at 10 weeks of age and challenged 94 days later. A. Sham-vaccinated mice experienced significant foot swelling compared to CHIKV/IRES-vaccinated mice. B. All vaccinated mice survived challenge while sham-vaccinated mice succumbed to infection by day 5. C. Sham-vaccinated animals experienced significant hyperthermia on day 2 and hypothermia on day 3, while vaccinated animals maintained relatively stable temperatures. D. There was no significant difference in weight change between the cohorts. *  = p<0.05; **  = p<0.001.

To evaluate the duration of immunity and protection after vaccination, cohorts of six A129 mice were immunized with CHIKV/IRES as described above, bled 21, 42, 56 and 92 days later, then challenged 94 days after vaccination. Similar to the results described above, no significant weight loss, footpad swelling, or other signs of disease were noted after vaccination compared to sham-vaccination (data not shown). Antibody PRNT_80_ titers prior to challenge were all ≥640. After challenge with 100 PFU of wt-CHIKV, vaccinated animals were significantly protected against foot swelling, fever and mortality (6/6 sham-vaccinated mice died by day 5, whereas all CHIKV/IRES-vaccinated mice survived until day 14 when the study was terminated)(Fig. 9A and B). The sham-vaccinated group experienced significant hyperthermia on day 2, followed by significant hypothermia on day 3 as the animals became moribund (Fig. 9C). There were no significant differences in weight change between the two cohorts (Fig. 9D).

### Immunogenicity and efficacy in adult C57BL/6 mice

To test the immunogenicity and efficacy of the CHIKV/IRES vaccine candidate compared with strain 181/25 in immunocompetent mice, cohorts (N = 9-10) of 3-week-old C57BL/6 mice were vaccinated SC with 10^5^ PFU, or with PBS as negative controls. Although 14-day-old and adult C57BL/6 mice develop lesions in the leg after footpad inoculation with wt CHIKV [37], [38], we used a more stringent, lethal intranasal (IN) challenge C57BL/6 model with the neuroadapted Ross CHIKV strain for efficacy testing [30]. Three weeks after infection, all mice were bled and Ab titers were measured using an 80% plaque reduction neutralization test (PRNT_80_). The mean Ab titers in response to strains CHIKV/IRES and 181/25 were nearly equal, with all animals exhibiting PRNT_80_ titers ≥20 (p>0.1; Table 1). The mice were then challenged IN with 10^6^ PFU of the Ross CHIKV strain. All vaccinated animals survived without any signs of disease (weight loss, temperature change, ruffling of fur or hunched posture) through day 14. One of ten sham-vaccinated mice died on day 9 and 6 died on day 10 after challenge. These results demonstrated the immunogenicity and significant efficacy of the CHIKV/IRES vaccine candidate in immunocompetent mice.

**Table 1:** Seroconversion of adult C57BL/6 mice after vaccination.

Vaccine	CHIKV/IRES	181/25	Sham
% Seroconversion	100	100	0
Mean PRNT_80_ titer	62	67	<20
Standard deviation	44	20	

9 animals per cohort, assayed 28 days after vaccination with 10^5^ PFU.

### Passive transfer of immune sera

**Figure 10:**
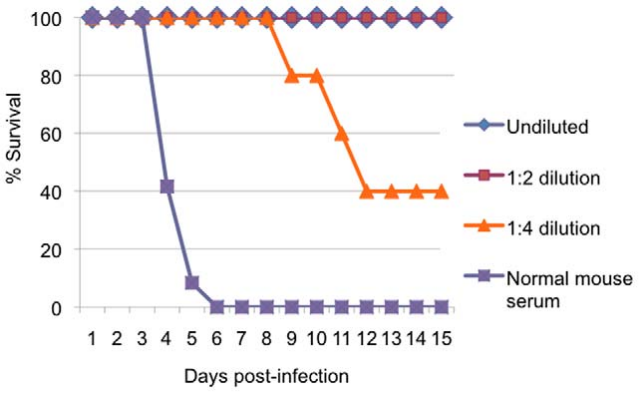
Protection of mice with passive transfer of immune serum. Survival of 10-week-old A129 mice after intraperitoneal inoculation of diluted or undiluted immune CD-1 mouse serum from animals vaccinated with CHIKV/IRES, and challenge with 100 PFU of wt-CHIKV. N = 5.

To confirm that neutralizing antibodies mediated protection of A129 mice from CHIKV challenge, pooled serum collected 21 days after immunization of A129 mice was inoculated intraperitoneally into naïve 6–7-week-old A129 mice (N = 5) either undiluted or at dilutions of 1:2 or 1:4; undiluted normal mouse serum was used as a negative control. Following challenge with 100 PFU of wt CHIKV, mortality was monitored for 15 days. All mice that received immune serum exhibited increased survival compared with those that received normal mouse serum (Kaplan Meier, p<0.001) and greater dilutions of the immune serum resulted in reduced survival (Fig. 10). These data indicate that neutralizing antibodies protected against fatal CHIK and indicate a correlation between Ab levels and protection.

### Mosquito infections in vivo

To confirm that the CHIKV/IRES strain was incapable of replicating in mosquitoes, cohorts of 20 adult female *Ae. albopictus*, a highly susceptible urban vector [20], were inoculated intrathoracically with ca. 1.0 µl of a 10^4^ PFU/ml suspension of either CHIKV/IRES or the wt LR strain. Intrathoracic infection was used rather than oral exposure because mosquitoes are uniformly susceptible to small CHIKV doses delivered via this route, whereas the oral portal of entry is less permissive even after large doses. After 7 days of incubation at 27°C, mosquitoes were triturated and serial 10-fold dilutions were tested for virus by inoculation of Vero cells followed by examination for cytopathic effects (CPE) through day 7. Mosquitoes inoculated with CHIKV/IRES as well as PBS-inoculated negative control mosquitoes produced no detectable CPE. In contrast, all 20 mosquitoes receiving wt CHIKV produced extensive CPE on the Vero cells. To ensure that temperature sensitive or host-restricted mutants were not generated following mosquito infection, RT-PCR targeting the 5′ end of the capsid gene was also used to detect viral RNA. No amplicons were detected from the CHIKV/IRES-infected mosquitoes by gel electrophoresis, whereas all mosquitoes injected with wt CHIKV produced strong bands of the expected size (Fig. S1).

## Discussion

Nearly 80 years after the introduction of the first vaccine against an arboviral disease, yellow fever [39], vaccination remains the most effective method to protect against arboviruses and many other infectious agents. In the case of CHIK, the 181/25 live-attenuated vaccine developed during the 1980s showed promise in preclinical studies [23] but was mildly reactogenic in human trials [24]. More recent vaccine development has focused on inactivated [26], DNA [27] or virus-like particle approaches [28]. However, in our opinion, the requirements for multiple doses administered over several weeks and/or the higher cost of such vaccines, as well as the probability that boosters will be required to maintain immunity, will limit their usefulness in the developing nations of Africa and Asia where CHIKV is endemic. We have therefore focused on live-attenuated vaccines to prevent both endemic and epidemic CHIK.

The maturity of reverse genetic technology has provided unprecedented opportunities for manipulation of the alphaviral genome to improve attenuation strategies [40]. Thus, unlike traditional attenuation approaches that rely on cell culture passages, which typically result in attenuation that depends only on small numbers of attenuating point mutations [41], alternative genetic strategies such as viral chimeras offer the promise of more stable attenuation [30], [42], [43], [44]. In addition to the risk of reactogenicity, attenuation based on small numbers of mutations can also result in residual alphavirus infectivity for mosquito vectors. This risk, which was underscored by the isolation of the TC-83 VEEV vaccine strain from mosquitoes in Louisiana during an equine vaccination campaign designed to control the 1971 epidemic [45], is especially high when a vaccine that relies on a small number of point mutations is used in a nonendemic location that could support a local transmission cycle.

To overcome the aforementioned limitations, we exploited the finding that the EMCV IRES sequence functions inefficiently for translation in insect cells [33], yet can replace the alphavirus subgenomic promoter to mediate translation of the structural polyprotein open reading frame from the genomic RNA in mammalian cells [31], [32]. The resultant CHIKV strain replicated efficiently in Vero cells, an acceptable vaccine substrate, and exhibited a stable plaque morphology and consensus genome sequence after 10 passages in this cell line. The CHIKV/IRES vaccine candidate was unable to replicate in mosquito cells or in the mosquito vector, *Ae. albopictus*, an important safety feature for an live arbovirus vaccine that may be administered to travelers or laboratory workers in nonendemic locations.

Attenuation, immunogenicity and efficacy of the CHIKV/IRES vaccine candidate was assessed alongside that of the 181/25 CHIKV strain, which is highly immunogenic in humans and other animals yet inadequately attenuated. The goal was to equal the immunogenicity of the 181/25 vaccine strain but to achieve greater attenuation. Using infant and adult immunocompetent [35] and interferon type I receptor-deficient mouse models [36], we demonstrated that CHIKV/IRES met both goals. As measured by survival and weight gain or maintenance, CHIKV/IRES was similarly or better attenuated than 181/25 in multiple mouse models, yet generated comparable neutralizing Ab titers and nearly complete protection against disease or mortality after CHIKV challenge. Immunity and protection were maintained for at least 3 months. Viremia after CHIKV/IRES vaccination was never detected in infant CD-1 mice, and was transiently present at a very low level in immunocompromised A129 mice, an important attenuation phenotype considering that viremia could potentially lead to mosquito infection. However, even in the unlikely event that vaccination of an immunocompromised human led to viremia, the mosquito-incompetent phenotype discussed above should prevent transmission. The only measure of efficacy for which strain 181/25 exhibited a slight superiority was in the prevention of footpad swelling post challenge; CHIKV/IRES-vaccinated A129 mice challenged with wt-CHIKV exhibited a greater mean of 0.15mm swelling versus only 0.09 mm for strain 181/25. However, both vaccines provided significant protection compared with sham-vaccination.

Splenic histopathology was used as a second measure of protection. Mice challenged with wt-CHIKV after sham vaccination developed severe necrosis along with a monocytoid infitrate. in contrast, the CHIKV/IRES vaccine induced no splenic histopathology and protected against splenic lesions upon challenge.

Previous attempts to use the EMCV IRES to generate an alphavirus vaccine used the VEEV live-attenuated vaccine strain TC-83 [30]. Although these studies succeeded in eliminating the ability of TC-83 to infect mosquito vectors, immunogenicity was reduced to the point where most vaccinated mice did not develop detectable neutralizing antibodies (although significant protection against challenge was still detected). In contrast, our CHIKV vaccine started with the genetic backbone of a virulent wt alphavirus (LR) (Fig. 1A), and robust immunogenicity was maintained despite strong attenuation. These results suggest that the IRES attenuation level may be optimal when applied to other wild-type alphavirus backbones. The application of this platform for attenuation is now being applied to Venezuelan, western, and eastern equine encephalitis viruses to test this hypothesis.

In summary, a novel CHIK vaccine candidate, CHIKV/IRES, was generated by manipulation of the structural protein expression of a wt-CHIKV strain via the EMCV IRES. This vaccine candidate exhibits a high degree of murine attenuation that is not dependent on an intact interferon type I response, yet is highly immunogenic and protects against CHIKV challenge. This promising vaccine candidate is being tested in nonhuman primates to determine if it is suitable for evaluation in humans.

## Materials and Methods

### Ethics statement

This study was carried out in strict accordance with the recommendations in the Guide for the Care and Use of Laboratory Animals of the National Institutes of Health. The protocol was approved by the Institutional Animal Care and Use Committees of the University of Texas Medical Branch or the University of Wisconsin.

### Cell cultures

Vero African green monkey kidney cells were obtained from the American Type Cell Culture (Bethesda, MD). The cells were maintained at 37°C in Eagles minimum essential media (MEM) supplemented with 10% fetal bovine serum (FBS), penicillin and streptomycin. C6/36 *Ae. albopictus* cells were also maintained in MEM containing 10% FBS at 32°C and supplemented with 10% tryptose phosphate.

### Production of plasmid and sequencing

The CHIKV cDNA clone containing the EMCV IRES with the subgenomic promoter ablated using 13 synonymous mutations (CHIKV/IRES) was produced using standard recombinant DNA techniques in which the infectious clone of La Reunion strain (LR) described previously was used as a template [34]. This CHIKV clone, a gift from Stephen Higgs, contains an SP6 bacteriophage promoter for transcription of RNA that is identical to genomic viral RNA. The IRES sequence was PCR amplified from a cDNA clone described previously [31]. The inactivation of the subgenomic promoter was done using site-specific mutagenesis. An intermediate construct encoding the 3′ end of the nsP4 gene through the subgenomic promoter was produced using PCR with high fidelity Phusion DNA polymerase from Finnzymes (Espoo, Finland). The resultant amplicon was cloned into a shuttle vector, prS2, and was sequenced using the BigDye kit (Applied Biosystems, Foster City, CA). The 5′ end of capsid gene from the LR strain was amplified using PCR with an overhang complementary to the IRES sequence. The IRES-containing and capsid fragments were then joined using fusion PCR, and this fragment was cloned back into the shuttle vector and resequenced. The IRES/Capsid fragment and the mutated subgenomic fragment were finally ligated together through the SpeI site introduced into both fragments. The completed insert was then cloned into the LR backbone and this final construct was completely sequenced.

### RNA transcriptions, transfections, and virus production

Large-scale plasmid purification was done using CsCl preparations. The purified DNA was then linearized using NotI restriction endonuclease (New England BioLabs, Ipswich, MA), and a small sample was analyzed on a 1.2% agarose gel to verify linearization. The remaining DNA was transcribed using an Ambion SP6 In vitro transcription kit. The RNA was quantified and used to electroporate Vero cells using a BTX ECM 830 electroporator. Briefly, two T-150 flasks containing 90% confluent Vero cells were trypsinized and washed 3 times in RNAse-free DPBS. The cells were resuspended in 700 µl of DPBS and 10 µg of RNA was added. The solution was placed in a 4mm cuvette and was pulsed 2 times at 250v for 10 msec at 1 sec intervals. The cells were then left at room temperature for 10 minutes before being plated in T-75 flasks. The virus was harvested at 24 hours post-electroporation and centrifuged at 771×g. Supernatant was collected and titered by plaque assay on Vero cells.

### Cell culture passages and replication curves

The CHIKV/IRES vaccine candidate was passaged in Vero and C6/36 cells to assess phenotypic and genetic stability. T-25 flasks were grown to 90-95% confluency, then were infected at a multiplicity (MOI) of 0.1 Vero PFU/cell. Following 30 h of incubation at 37°C or 32°C, respectively, the medium was diluted and used to infect another flask with a MOI of 0.1. Following 10 serial passages, consensus sequences were determined for both passaged populations and plaque-purified biological clones by RT-PCR amplification and amplicon sequencing. We also selected 10 well-isolated, random plaques, harvested virus using a plastic micropipette tip. The agar plug containing the plaque was placed in 300 µl of MEM containing 2% FBS and RNA was extracted using TRIzol LS (Invitrogen, Carlsbad, CA). RT-PCR and sequencing were performed as described above. Vero plaque sizes were measured and compared to assess stability.

Replication kinetics was measured in 35 mm 6-well plates with duplicates for each virus tested. The wells were seeded to a confluency of 95% using Vero cells. Media was removed and they were infected at an MOI of .1 for one hour. Then 2.1 ml of DMEM containing 5% FBS was added. A 0 time point was immediately removed (100 µl). At each of the remaining time points 12, 24, 36 and 48 100 µl was removed and replaced. The samples were tittered as described above.

### Virus and antibody titers

Depending on containment requirements and sensitivity needs, virus stocks and experimental samples were titered by plaque assay as previously described [46] or were estimated using quantitative real-time PCR with dilutions of virus to generate standard curves from which PFU titers could be extrapolated. This assay used primers (5′-GAYCCCGACTCAACCATCCT-3′) and (5′-CATMGGGCARACGCACTGGTA-3′) and the probe (5′-AGYGCGCCAGCAAGGAGGAKGATGT-3′) which contained the dye FAM. Ab titers were measured using plaque reduction neutralization tests with 80% reduction endpoints [46].

### RNA replication

Vero cells were infected on 35 mm^2^ 6 well plates at an MOI of 20. The media was removed 18 hours after infection and replaced with .8 ml of complete media with 1 µg/ml of actinomycin D from Sigma, and 20 µCI of [5,6-^3^H] uridine from Moravak Biochemicals (Brea, CA.). The cells were then incubated for 4 hours and RNA is removed by TRIzol extraction. The RNA was placed into a sodium phosphate buffer containing DMSO and glyoxal at 50°C for 1 hour. The RNA was loaded into a 1% agarose gel and run at 150 v for 3–4 hours. The gel was then washed twice in methanol for 30 minutes. Then a 2.5% PPO and methanol solution was placed with the gel overnight. The gel was washed with DI water to precipitate the PPO and the gel was then dried. The gel is then placed with X-OMAT AR film (Kodak), at −80°C for 8 hours.

### Animal studies

Five-to-seven-day-old CD1 outbred mice [35] were obtained from Charles River (Wilmington, MA). These animals were infected subcutaneously (SC) with 10^5^ PFU and were serially sacrificed on days 2, 4, 6, 8, and 10. Blood, brain, and hind femoral tissues were collected for assays of virus content. C57BL/6 mice were obtained from Jackson labs (Bar Harbor, ME) and used in challenge experiments as described previously [30]. Briefly, the animals were infected SC at 3 weeks of age with 10^5^ PFU in the hind leg and observed for signs of illness for 21 days. Then, they were challenged intranasally (IN) with 10^6.5^ PFU of the neuroadapted Ross CHIKV strain. The animals were observed daily for illness and were sacrificed when they became moribund.

A129 mice were bred at the University of Wisconsin from a breeding pair obtained from B & K ltd. Grimston, England. Animals 3 or 10 weeks of age, were infected with 1x10^4^ PFU of vaccine strains ID in the left rear footpad. Footpad measurements were taken 48 hours post vaccination with a caliper as the vertical height of the hind feet at the balls. The animals were maintained for 38 days and bled on days 21 and 35. These animals were then challenged with 100 PFU of wt CHIKV and were monitored for morbidity and mortality. All animals were euthanized by CO_2_ overdose if they became moribund. A129 animals were used for a longitudinal study of protection in which they were challenged with 100 PFU ID of wt-CHIKV 94 days after being vaccinated.

Tissues were fixed in 10% neutral buffered formalin (RICCA Chemical Company, Arlington, TX.). Bone tissue was decalcified overnight using fixative/decalcifier (VWR International, Radnar, PA.). Tissue was then embedded in paraffin wax and 5 um sections were cut for analysis. Sections for hematoxylin and eosin staining were deparaffinized in Xylene for 15 minutes. Sections were then rehydrated in ethanol and ethanol/water mixtures as follows: 100% ethanol for 9 minutes, 95% ethanol/5% deionized water for 3 minutes, 80% ethanol/20% deionized water for 5 minutes. Sections were then stained with hematoxylin (Richard-Allan Scientific) for 3 minutes and then rinsed with deionized water. Sections were then rinsed in tap water for 5 minutes and placed in Clarifier I (Richard-Allan Scientific, Kalamazoo, MI.) for 5 minutes. Sections were then rinsed in tap water for 2 minutes and then in deionized water for 2 minutes. Sections were then stained in eosin (Richard-Allan Scientific) for 30 seconds. They were then dehydrated as follows: 95% ethanol/5% deionized water for 15 minutes, 100% ethanol for 15 minutes and then Xylene (Richard-Allan Scientific) for 15 minutes. Cover slips were applied to slides using Permount (Fisher Scientific) and dried overnight. Deparaffinizing and hematoxylin-eosin staining was performed on the Varistain Gemini ES (Shandon, Thermo Fisher Scientific).

All animal studies were approved by the UTMB and/or the Univ. Wisconsin Institutional Animal Care and Use Committee.

### Mosquito infections

An *Ae. albopictus* colony established in 2003 from mosquitoes collected in Galveston, TX was used for these experiments. This species was selected because it is highly susceptible to the LR CHIKV strain [20]. Adult female mosquitoes collected 3–4 days post-eclosion were anesthetized using a chill table (Bioquip, Rancho Dominguez, CA) and were then injected intrathoracically with ca. 1.0 µL of a 10^4^ Vero PFU/ml virus stock. The mosquitoes were incubated for 7 days at 27°C with 10% sucrose provided *ad libitum*. The mosquitoes were then frozen and triturated in MEM containing 2% FBS and fungicide using a Tissuelyser II (Qiagen, Venlo, Netherlands) for 2 min. Following centrifugation for 10 minutes at 10,000×G, the supernatant was plated on Vero cells using 96 well plates. The cells were infected for 1 hour at 37°C and then covered with 2% FBS containing MEM and allowed to incubate for 48 hr to measure CPE.

### RT-PCR

RNA was collected through Qiagen RNeasy columns (Qiagen, Venlo, Netherlands) or TRIzol LS (Invitrogen) using the manufacturer's protocols. 130 µl of sample were taken from the mosquito homogenates and the RNA was collected. The RNA was then amplified via RT-PCR using a Titan single step RT-PCR kit (Roche, Basel, Switzerland). The primers used to amplify annealed to the 5′end of capsid, 5′-TGGCCTTTAAGCGGTC-3′ and 5′-TATGGTCTTGTGGCTTTATAGAC-3′.

### Statistics

Student's T-tests were performed using Excel (Microsoft, Redmond, WA). ANOVA tests were performed using SPSS v18 (IBM Corporation, Somers, NY). Kaplan-Meier tests were performed using Prism 5 (GraphPad Software, La Jolla, CA). P-values <0.05 were considered significant. Negative data points were counted at one-half of the corresponding limit of detection for statistical analyses.

## Supporting Information

Figure S1 **Detection of CHIKV RNA in intrathoracically inoculated mosquitoes using RT-PCR.** Mosquitoes were injected with ca. 1 µl of a 10^4^ PFU/ml virus stock or sham inoculated with PBS, and harvested 7 days later. Viral RNA was extracted and subjected to RT-PCR targeting the capsid protein gene. PCR products were electrophoresed on a 1% agarose gel and DNA was stained with ethidium bromide. Arrow shows the expected amplicon size of 565 bp.
(TIF)
