# Supplementary figures and images for: Novel Chikungunya Vaccine Candidate with an IRES-Based Attenuation and Host Range Alteration Mechanism
Source: PLoS Pathog. 2011 Jul 28;7(7):e1002142. doi: 10.1371/journal.ppat.1002142 (PMC3145802; doi:10.1371/journal.ppat.1002142)

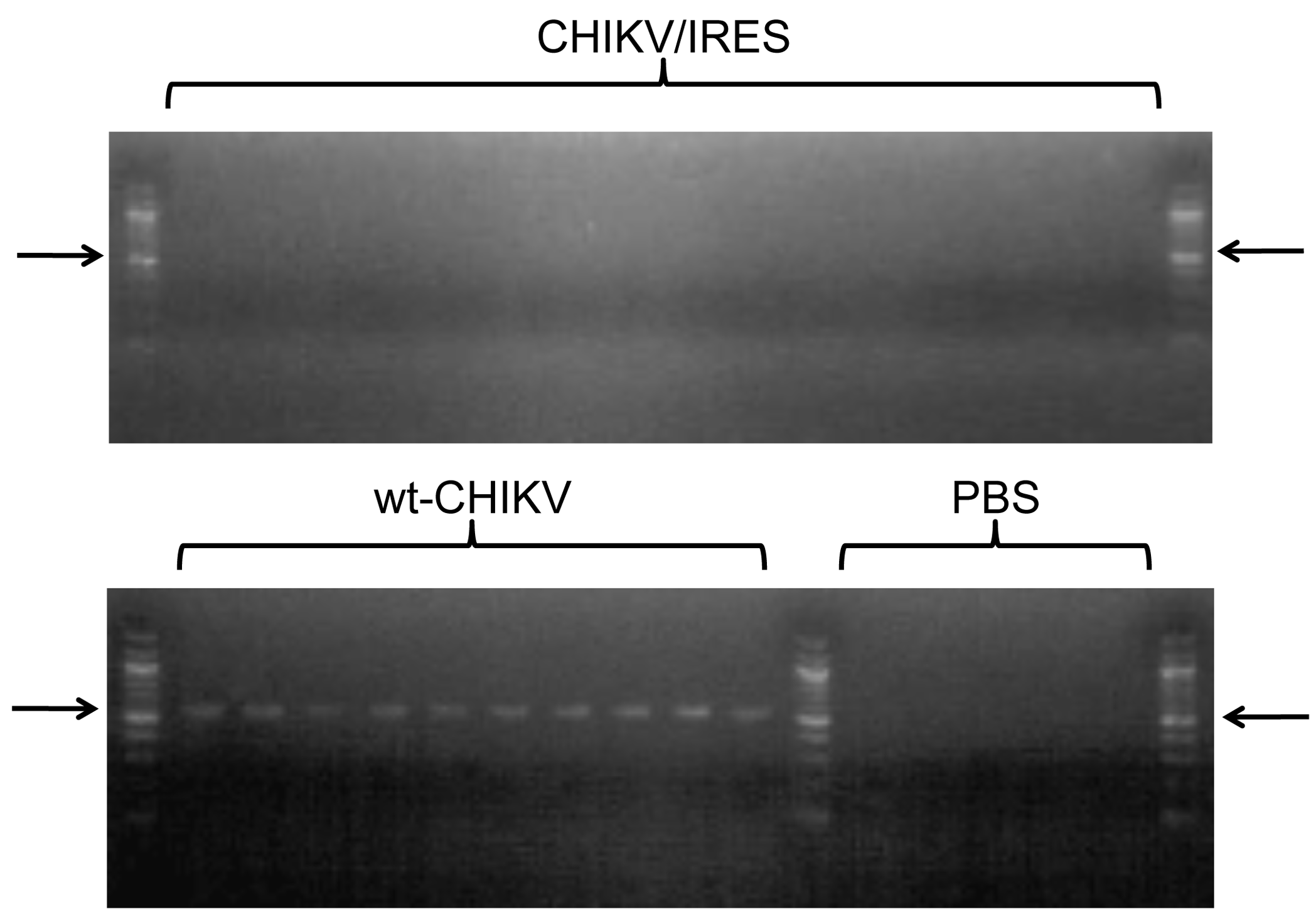

Supplement: Figure S1 — Detection of CHIKV RNA in intrathoracically inoculated mosquitoes using RT-PCR. Mosquitoes were injected with ca. 1 µl of a 104 PFU/ml virus stock or sham inoculated with PBS, and harvested 7 days later. Viral RNA was extracted and subjected to RT-PCR targeting the capsid protein gene. PCR products were electrophoresed on a 1% agarose gel and DNA was stained with ethidium bromide. Arrow shows the expected amplicon size of 565 bp. (TIF) [file ppat.1002142.s001.tif]
